# E-commerce platform financing versus trade credit financing: Financing mode selection for online retailer considering live-stream selling in China

**DOI:** 10.3389/fpsyg.2022.1078369

**Published:** 2023-01-10

**Authors:** Shuai Huang, Bingzhi Du, Zhi-Ping Fan, Zhixi Liu

**Affiliations:** ^1^Department of Management Science and Engineering, Business School, Qingdao University, Qingdao, China; ^2^Department of Information Management and Decision Sciences, School of Business Administration, Northeastern University, Shenyang, China; ^3^National Frontiers Science Center for Industrial Intelligence and Systems Optimization, Northeastern University, Shenyang, China

**Keywords:** capital-constrained, e-commerce platform financing, online retailer, trade credit financing, live-stream selling

## Abstract

**Introduction:**

The rise of live-stream selling has made the e-commerce platform attractive to many small and medium-sized retailers that are often faced with capital constraints. The choice between the e-commerce platform financing (EPF) and trade credit financing (TCF) for the capital-constrained e-retailers engaging in live-stream selling is particularly important problem.

**Methods:**

This paper considers a supply chain made up of a manufacturer, an e-commerce platform that offers live-stream selling service to consumers and an online retailer with capital constraint. We, respectively, investigate the optimal decisions of the supply chain enterprises under EPF and TCF modes based on Stackelberg game models and optimization theories.

**Results:**

We compare the profits of supply chain firms under different cases and obtain some important conclusions through theoretical and numerical analysis.

**Discussion:**

First, when the e-commerce platform’s commission rate is low enough, the retailer’s ordering quantity is, under EPF mode, greater than that evidenced without capital constraint. In addition, when the retailer’s marginal profit is high and the e-commerce platform’s commission rate is low, the online retailer should choose EPF mode; in other instances, TCF is its optimal choice. Second, the e-commerce platform can obtain the highest profit under EPF mode, while TCF mode will bring the highest profit to the manufacturer. Third, when the platform’s commission rate is below a certain threshold, the profit of the entire supply chain under EPF mode is larger than that of well-funded supply chain, but TCF mode cannot. Finally, we also find there exists the access threshold about the live-stream selling. Only when the commission rate is relatively high, the e-commerce platform should offers live-stream service to consumers and the live-stream investment is the highest under EPF mode.

## 1. Introduction

As a result of the rapid development of e-commerce, many retailers now sell their products through e-commerce platform, and this is attested to by the fact that the total sales of Chinese e-commerce reached 4.28 trillion dollars in 2020, a significant increase of 27.6% compared to the previous year (Tunca and Zhu, 2018; He et al., 2019; Li and Jiang, 2019; Zhang et al., 2020). Recently, a new sales mode called live-stream shopping, has become increasingly popular and has been adopted by many giant e-commerce retailers, such as Tmall and JD (Hao and Yang, 2022; Pan et al., 2022). Compared to the traditional online channel, the streamer can sell and introduce products through real-time interactions with consumers in a live-stream channel. Therefore, consumers can obtain more detailed information, which can build customer trust (Hilvert-Bruce et al., 2018; Wongkitrungrueng and Assarut, 2018; Park and Lin, 2020). In practice, many consumers prefer to purchase through the live-stream channel due to the authenticity, visualization and real-time interactivity, which can significantly increase sales (Hao and Yang, 2022; Pan et al., 2022). For example, Jiaqi Li, a famous streamer on TABOBAO, could sell 15,000 lipsticks in only 5 min through live streaming shopping (Zhang et al., 2022).

In comparison with traditional retailers, most online retailers are small and medium-sized enterprises, who are generally confronted by the challenge of insufficient funds (Dada and Hu, 2008; Kouvelis and Zhao, 2016; Huang et al., 2019; Wang et al., 2019). Supply chain financing is the most effective method through which capital-constrained firms can obtain funds (Levaggi, 1999; Che and Gale, 2000; Zhou et al., 2017; Jin et al., 2018). It is the most common financing mode that the bank offers to an online retailer afflicted by capital constraint (Buzacott and Zhang, 2004; Kouvelis and Zhao, 2016; Yan et al., 2016). However, most small and medium retailers find it difficult to obtain funds from the bank because online retailers lack tangible internal resources (Tang et al., 2018). In addition to the bank financing (BF) mode, the online retailer can often accept financing service from the manufacturer, in the form of the trade credit financing (TCF) mode (Chen, 2015; Peura et al., 2017).

In recent years, more e-commerce platforms have sought to provide financing services to cooperative consumers and enterprises with capital constraint, with examples including JD.com and Alibaba (Tunca and Zhu, 2018; Wang et al., 2019). E-commerce platform financing (EPF) has become a new type of financing mode that is favored by a large number of small and medium-sized enterprises in the e-commerce supply chain (Tunca and Zhu, 2018; Gupta and Chen, 2020). When the manufacturer and the e-commerce platform can both offer a financing service to the online retailer with capital constraint, it is worthwhile to consider how the retailer chose the financing mode and what the response strategies of the manufacturer and e-commerce platform will be in various cases. Research of the comparative analysis between TCF and EPF is necessary and important, but is rarely evidenced in existing literatures. In this paper, we obtain the optimal decisions of e-commerce supply chain partners in TCF and EPF, and compare the value of the two modes for the supply chain. More specifically, we focus on the following three questions:

i. How do supply chain enterprises determine their optimal operational strategies under TCF and EPF modes?

ii. Which factors affect the financing mode preference of supply chain enterprises?

iii. Which kind of financing mode benefits the supply chain? Is it TCF or EPF?

This paper aims to address these questions by performing a relevant theoretical analysis with the intention of extracting valuable conclusions and important managerial implications that can then be applied to financing mode choices and the operational decisions of e-commerce supply chain that have capital constraints.

In this study, we consider a supply chain that consists of a manufacturer, an e-commerce platform and an online retailer with capital constraint. We consider that the e-commerce platform provides the live-stream selling service to consumers. Meanwhile the manufacturer and the e-commerce platform can both provide a financing service to the online retailer with a certain interest rate, specifically the TCF and EPF mode. First, we investigate the e-commerce platform’s optimal live-stream investment and the retailer’s optimal ordering quantity without capital constraint. We also seek to determine the manufacturer’s optimal interest rate or e-commerce platform, along with (respectively) the e-commerce platform’s optimal live-stream investment and the retailer’s optimal ordering quantity under two financing modes. Second, in analyzing the value of TCF and EPF, we compare the profits of supply chain partners in different cases. In addition, we examine how the e-commerce platform’s commission rate and the online retailer’s marginal profit impact on the decisions and profits of supply chain firms. Theoretical and numerical analysis enables us to obtain some useful conclusions and extract managerial implications.

This paper contributes to the literature in two key ways. First, in engaging from the perspective of e-commerce platform’s finance and live-stream role, we study the financing mode choice between EPF and TCF with reference to the online retailer with capital constraint, which has been insufficiently explored by existing studies. Our study compensates for gaps within supply chain finance to some extent. Second, through theoretical and numerical analysis, we obtain the specific conditions under which the online retailer chooses the optimal financing mode and thereby obtain practical managerial insights into the practical operational decisions of an e-commerce supply chain that has capital constraint.

The remainder of the paper takes the following form. Section 2 reviews the related literature before the following Section 3 describes the problem and explains the notations and assumptions in our models. Section 4 then obtains the optimal financing and operational decisions of the supply chain firms under the benchmark scenario, the TCF and EPF modes. Section 5 compares TCF and EPF by undertaking some theoretical analysis and offering a series of numerical examples before the discussion of Section 6. Section 7 concludes and suggests future research directions.

## 2. Literature review

Our research is closely related to two streams, specifically the operational strategy of the capital-constrained supply chain under TCF and platform financing.

In recent years, research into the operational strategy of the capital-constrained supply chain under TCF has attracted the attention of many scholars (Burkart and Ellingsen, 2004; Cai et al., 2014; Zhang et al., 2016; Yang et al., 2017; Cao and Yu, 2018; Wang et al., 2018; Fu et al., 2021). More specifically, Jing et al. (2012) obtain the optimal strategies of supply chain enterprises under TCF and BF modes by solving the game models. They find that the supplier will give a lower wholesale price to encourage the capital-constrained retailer to choose TCF, as it can benefit the supplier. Chen and Wang (2012) consider a capital-constrained retailer with limited liability and analyze the impact of TCF on supply chain performance. Their main inference is that TCF can create added-value for the capital-constrained supply chain. Chen (2015) highlights that TCF can improve the profits of supply chain members. He also finds that it better integrates the channel than BF since TCF is a kind of internal financing mode. Lee et al. (2018) study the impact of TCF on enterprise performance. They find that the supplier with smaller market share will offer more TCF, because this can strengthen its competitive advantage. Zhang et al. (2018) consider customer balking behavior, market information asymmetry and sharing, and this enables them to get the optimal decisions of the supplier and retailer in each scenario. Zhan et al. (2019) propose an innovative TCF with rebate contract in a capital-constrained supply chain and study the equilibrium selection between the innovative TCF and traditional TCF modes. Wu et al. (2019) propose a competitive supply chain that consists of a manufacturer, a dominant retailer and a weak retailer with capital constraint. Their study shows that TCF that the manufacturer offers to the weak retailer can benefit the weak retailer and damage its dominant counterparts. Nigro et al. (2021) consider a capital-constrained supply chain that consists of a supplier and a retailer, and study the role of credit rating and retailer effort on optimal contracts under internal and external financing modes. These related literatures about TCF provide valuable conclusions and managerial implications that extend to the operational management of the capital-constrained supply chain.

In the past few years, the platform financing has attracted wide attention in the research of supply chain finance (Gao et al., 2018; Liu et al., 2019; Jena et al., 2022; Wang et al., 2022; Yan et al., 2022). For instance, Tunca and Zhu (2018) analyze the role of buyer intermediation in supplier financing and demonstrate that buyer intermediation financing can improve channel performance and increase enterprise profits. Wang et al. (2019) study the financing mode choice between EPF and BF for the online retailer with capital constraint. They conclude that the active EPF can achieve online supply chain coordination, and contribute greater order to the quantity and profit of supply chain partners. Gupta and Chen (2020) propose an e-commerce supply chain that consists of a supplier with capital constraint and an online retailer (web platform). They obtain the equilibrium terms of the loan that the retailer offers to the supplier, and compare the platform and bank financing. Chang et al. (2022) consider a supply chain that consists of an online retailer with capital constraint and an e-commerce platform. They compare the platform and bank financing modes and find that when working capital is low, platform direct financing has no advantage over bank financing. These related literatures about platform financing provide valuable conclusions and managerial implications that have a clear application to the operational management of online supply chains with capital constraint.

Compared with the supply chain financing in traditional offline and online channels, the online supply chain financing of live-stream selling channel have special characteristics. First, it is *the interaction among live-stream service, product demand and financing amount*. In live-stream selling channel, consumers can get more detailed information about the products and communicate with the streamers and other consumers in real-time, which can have a positive impact on the consumer’s purchasing behavior. Because of the raise of product demand, the online retailer will have a greater funding requirement. Therefore, the interaction among three factors will impact the operational and financing decisions of online supply chain. Second, it is *the combined effects of multiple services provided by the e-commerce platform*. Under EPF mode, the e-commerce platform also provide live-stream marketing service and financing service besides platform sales service for the online retailer. How does the multiple services impact the financing preference of the online retailer? This is also an urgent need to resolve. However, there are few studies that investigate the choice of financing modes about TCF and EPF under the live-stream selling background.

In providing a study of this kind, this paper will consider the situation of the online retailer with capital constraints in an e-commerce supply chain, and will investigate how TCF and EPF impact on supply chain performance and enterprise preference.

## 3. Notations and assumptions

This paper considers an e-commerce supply chain that consists of a manufacturer, an e-commerce platform and an online retailer with capital constraint. The online retailer orders the product $q_{i}$ from the manufacturer at a certain wholesale price w and sells the product to consumers at a certain retail price p through the live-stream selling channel providing by the e-commerce platform. And the online retailer needs to pay the commission to the e-commerce platform after the sales at a certain commission rate λ. Meanwhile the e-commerce platform provides live-stream selling service to the consumers in our study.

**Figure 1:**
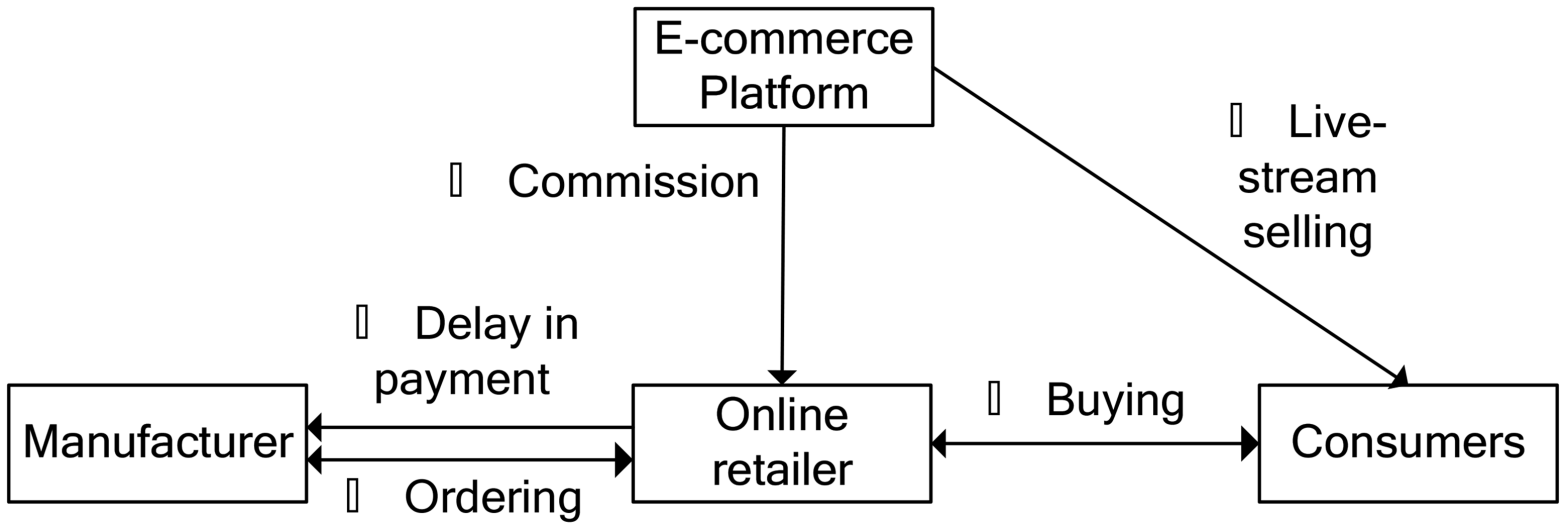
Trade credit financing.

**Figure 2:**
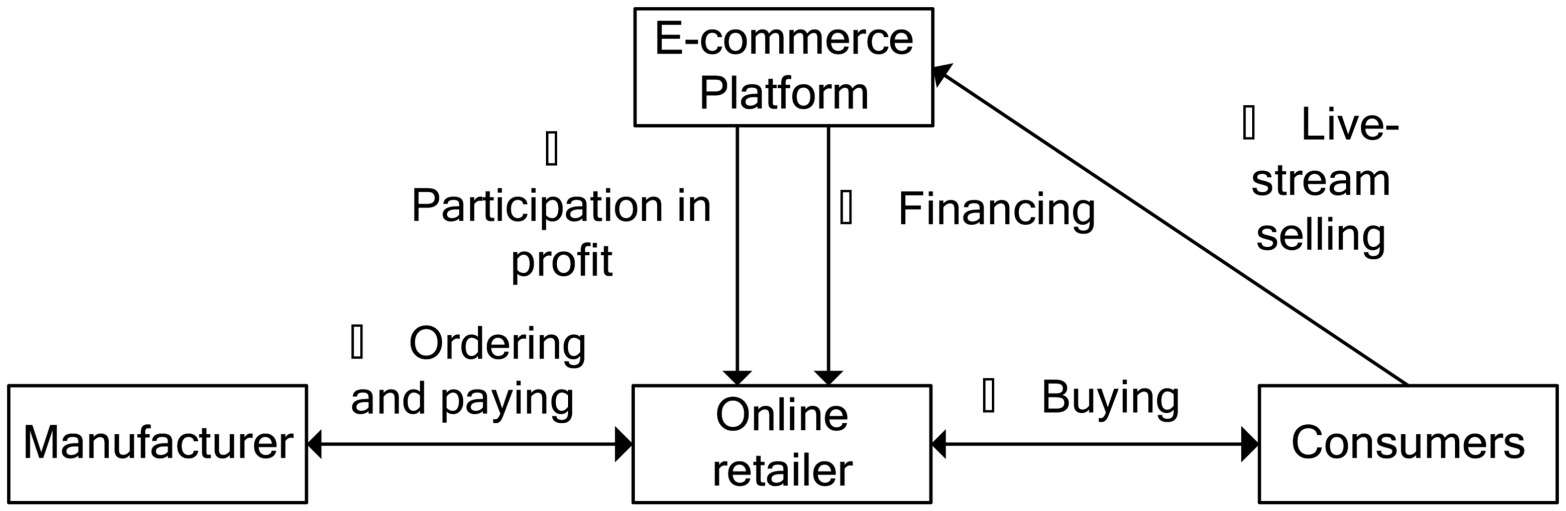
E-commerce platform financing.

We consider that the retailer is capital-constrained at the beginning of the sales season, and that the manufacturer and the e-commerce platform can, in the form of the TCF and EPF modes, both provide financing service to the online retailer with a certain interest rate $r_{m}$ and $r_{e}$. This arrangement is shown in Figures 1, 2.

The sequence of events related to the TCF shown in Figure 1 can be explained as follows:

**①** at the beginning of the sales season, the manufacturer sets its interest rate $r_{m}$ and offers a financing service to the capital-constrained retailer, and the retailer then orders $q_{m}$ from the manufacturer at a certain wholesale price w; **②** the consumers can obtain much information about the products through the live-stream channel, and the e-commerce platform decides the live-stream investment $t_{m}$; **③** the consumers buys products from the retailer at a certain retail price p, and pay the e-commerce platform for products; **④** the e-commerce platform pays the retailer for goods after deducting the retailer’s commission; **⑤** at the end of the sales season, the retailer is required to repay the ordering cost and interest to the manufacturer.

With regard to the EPF shown in Figure 2, the sequence of events can be explained as follows:

**①** at the beginning of the sales season, the e-commerce platform sets its interest rate $r_{e}$ and lends money to the capital-constrained retailer; **②** the retailer orders $q_{e}$ from the manufacturer and provides payment in return; **③** the consumers can obtain much information about the products through the live-stream channel, and the e-commerce platform decides the live-stream investment $t_{e}$; **④** the consumer buys products from the retailer at a certain retail price p, and need to repay the payment for goods to the e-commerce platform; **⑤** the e-commerce platform deducts the retailer’s commission and loan principal (with interest), before passing the surplus profit to the online retailer.

Some notations will now be defined and explained in order to help describe and analyze the model. In our study, the indexes i=o,m,e respectively denote the benchmark scenario (the online retailer has no capital constraint) and the TCF and EPF modes. In addition, M, E and R respectively denote the manufacturer, e-commerce platform and retailer.

*Decision variable.*

$q_{i}$: The online retailer’s ordering quantity;

$t_{i}$: The live-stream investment of e-commerce platform;

$r_{m}$: The manufacturer’s interest rate under the TCF mode, $r_{m}\geq 0$;

$r_{e}$: The e-commerce platform’s interest rate under the EPF mode, $r_{e}\geq 0$;

*General parameters.*

c: The manufacturer’s unit production cost;

w: The manufacturer’s unit wholesale price;

p: The online retailer’s unit retail price;

λ: The commission rate of the e-commerce platform, 0<λ<1;

φ: The cost coefficient of live-stream for the e-commerce platform, 0<φ<1;

a: The potential demand in the market;

θ: The preference coefficient of consumers for the live-stream investment;

$d_{i}$: The market demand for a product;

$\Delta^{M}$: The marginal profit of the manufacturer, $\Delta^{M}=w-c$;

$\Delta^{R}$: The marginal profit of the online retailer, $\Delta^{R}=p\left(1-\lambda \right)-w$.

We assume that the market demand is $d_{i}=a+\theta t_{i}+\varepsilon ,$ where ε follows uniform distribution on $\left[-\left(a+\theta t_{i}\right),\;a+\theta t_{i}\right]$ (see Hilvert-Bruce et al., 2018; Wongkitrungrueng and Assarut, 2018; Park and Lin, 2020). The existing literature finds that the live-stream cost increases in the live-stream investment and sales volume, and we therefore assume that the live-stream cost is $\varphi t_{i}p\min\left(q_{i},\;d_{i}\right)$ (see Hao and Yang, 2022; Pan et al., 2022; Zhang et al., 2022). Without loss of generality, we have c<w<p(1−λ) and $\lambda >\varphi t_{i}$, which can ensure that the manufacturer, e-commerce platform and online retailer obtain the positive profits (Lou and Wang, 2013; Wang et al., 2014; Tsao, 2017). In this study we assume that each of these agents are risk-neutral and rational and operate in a perfectly competitive capital market. In addition, it is also assumed that information is shared symmetrically among enterprises (Dada and Hu, 2008; Lou and Wang, 2013; Kouvelis and Zhao, 2016; Tsao, 2017).

## 4. Optimal decisions in different scenarios

In this section we investigate the optimal financing interest rate, live-stream investment and ordering quantity of the manufacturer, e-commerce platform and online retailer, and make (respective) reference to the benchmark scenario and the TCF and EPF modes.

### 4.1. Benchmark scenario

In order to facilitate the analysis of TCF and EPF, it is first necessary to obtain the optimal strategies of unconstrained supply chain partners, as this can help to establish the benchmark scenario. When the online retailer has sufficient funds at the beginning of the sales season, the e-commerce platform initially provides the live-stream investment $t_{o}$ before the online retailer then determines its ordering quantity $q_{o}$ and pays the manufacturer $wq_{o}.$ At the end of the sales season, the e-commerce platform can obtain the commission $\lambda p\min\left(q_{o},\;d_{o}\right)$ from the online retailer. In the meantime, the online retailer yields the remaining profit $\left(1-\lambda \right)p\min\left(q_{o},\;d_{o}\right)$. We therefore express the optimization problems of the e-commerce platform and well-funded retailer in Eqs 1, 2:


(1) $$\underset{t_{o}}{\max}E\left[\Pi_{o}^{E}\left(t_{o}\right)\right]=\underset{t_{o}}{\max}E\left[p\left(\lambda -\varphi t_{o}\right)\min\left(q_{o},\;d_{o}\right)\right]\;,$$

(2) $$\underset{q_{o}}{\max}E\left[\Pi_{o}^{R}\left(q_{o}\right)\right]=\underset{q_{o}}{\max}\left\{E\left[p\left(1-\lambda \right)\min\left(q_{o},\;d_{o}\right)\right]-wq_{o}\right\}.$$

Eqs 1, 2 can be changed as follows (see Dada and Hu, 2008):


(3) $$\underset{t_{o}}{\max}E\left[\Pi_{o}^{E}\left(t_{o}\right)\right]=\underset{t_{o}}{\max}p\left(\lambda -\varphi t_{o}\right)\left[q_{o}-\frac{q_{o}^{2}}{4\left(a+\theta t_{o}\right)}\right]\;,$$

(4) $$\underset{q_{o}}{\max}E\left[\Pi_{o}^{R}\left(q_{o}\right)\right]=\underset{q_{o}}{\max}\left\{p\left(1-\lambda \right)\left[q_{o}-\frac{q_{o}^{2}}{4\left(a+\theta t_{o}\right)}\right]-wq_{o}\right\}\;.$$

Eqs 3, 4 help to establish the following proposition. All proofs are in the Supplementry Appendix.

Proposition 1.

When the online retailer has sufficient funds, the e-commerce platform’s optimal live-stream investment satisfies $t_{o}^{*}=\frac{\theta \lambda -a\varphi }{2\theta \varphi }$, and the well-funded retailer’s optimal ordering quantity satisfies $q_{o}^{*}=\frac{\left(a\varphi +\theta \lambda \right)\Delta^{R}}{\varphi p\left(1-\lambda \right)}$.

In accordance with Proposition 1, we have the following lemma and corollaries.

Lemma 1.

Under the benchmark scenario, there exists a lower limit threshold $\lambda_{o}=\frac{a\varphi }{\theta }$ when the e-commerce platform offers the live-stream selling service to consumers.

Lemma 1 shows that when the commission rate exceeds a certain threshold, the e-commerce platform may offer the live-stream selling service to consumers. The reason for this is that, when the commission rate is low, the e-commerce platform will, as a result of live-stream cost, incur a loss.

Corollary 1.

$\frac{\partial t_{o}^{*}}{\partial \theta }>0$, $\frac{\partial t_{o}^{*}}{\partial \lambda }>0$ and $\frac{\partial t_{o}^{*}}{\partial \varphi }<0$.

Corollary 1 indicates that the e-commerce platform will enhance live-stream investment if there is a high consumers’ preference coefficient for the live-stream investment or a high e-commerce platform’s commission rate. The same will also apply if the live-stream cost coefficient is low. The reason for this is that there is a positive association between the consumers’ preference coefficient, the e-commerce platform’s commission rate and the e-commerce platform’s profitability – when the first two increase, so does this the third, and this helps to determine a larger live-stream investment. Conversely, increases within live-stream cost coefficient will raise the risk of loss to the e-commerce platform, and this will produce a smaller live-stream investment.

Corollary 2.

(i) $\frac{\partial q_{o}^{*}}{\partial \theta }>0$, $\frac{\partial q_{o}^{*}}{\partial \varphi }<0$; (ii) when $\lambda <1-\sqrt{\frac{\theta w+a\varphi w}{\theta p}}$, then $\frac{\partial q_{o}^{*}}{\partial \lambda }>0$, otherwise, we have $\frac{\partial q_{o}^{*}}{\partial \lambda }\leq 0$.

When the consumers’ preference coefficient for live-stream investment is high, the demand is substantial, and this can stimulate the online retailer to order in great quantities. Corollary 1 establishes that increases in the live-stream cost coefficient will shorten the e-commerce platform’s live-stream investment and produce diminished, with the consequence that the online retailer will order in lower quantities.

In addition, when the e-commerce platform’s commission rate falls below a certain threshold, increases in the commission rate can raise the platform’s live-stream investment and demand, and this can in turn result in the online retailer ordering in greater quantities. However, when the e-commerce platform’s commission rate exceeds the threshold, the raised sales profit struggles to offset the online retailer’s increased commission, and accordingly its ordering quantity decreases in accordance with the e-commerce platform’s commission rate.

### 4.2. Trade credit financing mode

In this mode, the manufacturer decides the financing interest rate for the online retailer at the beginning of the sales season $r_{m}$, the e-commerce platform then gives the live-stream investment $t_{m}$ and the online retailer finally determines its ordering quantity $q_{m}$. At the end of the sales season, the e-commerce platform can obtain the commission $\lambda p\min\left(q_{m},\;d_{m}\right)$ and take on an expected cost for the live-stream selling $\varphi t_{m}p\min\left(q_{m},\;d_{m}\right).$ The online retailer, meanwhile, yields the remaining profit $\left(1-\lambda \right)p\min\left(q_{o},\;d_{o}\right)$ and it needs to pay the manufacturer $w\left(1+r_{m}\right)q_{m}$. We therefore express the optimization problems of the manufacturer, e-commerce platform and well-funded retailer in Eqs 5–7:


(5) $$\underset{r_{m}}{\max}E\left[\Pi_{m}^{M}\left(r_{m}\right)\right]=\underset{t_{m}}{\max}E\left[w\left(1+r_{m}\right)q_{m}-cq_{m}\right]\;,$$

(6) $$\underset{t_{m}}{\max}E\left[\Pi_{m}^{E}\left(t_{m}\right)\right]=\underset{t_{m}}{\max}E\left[p\left(\lambda -\varphi t_{m}\right)\min\left(q_{m},\;d_{m}\right)\right],$$

(7) $$\underset{q_{m}}{\max}E\left[\Pi_{m}^{R}\left(q_{m}\right)\right]=\underset{q_{m}}{\max}\left\{\begin{array}{l} E\left[p\left(1-\lambda \right)\min\left(q_{m},\;d_{m}\right)\right]\\ -w\left(1+r_{m}\right)q_{m} \end{array}\right\}.$$

According to Eqs 5–7, we have the following proposition.

Proposition 2.

Under the TCF mode:

(i) When $\Delta^{M}<\Delta^{R}$, the manufacturer’s optimal interest rate is $r_{m}^{*}=\frac{\Delta^{R}-\Delta^{M}}{2w}$, the e-commerce platform’s optimal live-stream investment is $t_{m}^{*}=\frac{\theta \lambda -a\varphi }{2\theta \varphi }$, and the online retailer’s optimal ordering quantity is $q_{m}^{*}=\frac{\left(a\varphi +\theta \lambda \right)\left(\Delta^{R}+\Delta^{M}\right)}{2\varphi p\left(1-\lambda \right)}$;

(ii) When $\Delta^{M}\geq \Delta^{R}$, the manufacturer’s optimal interest rate is $r_{m}^{*}=0$, the e-commerce platform’s optimal live-stream investment is $t_{m}^{*}=\frac{\theta \lambda -a\varphi }{2\theta \varphi }$, and the online retailer’s optimal ordering quantity is $q_{m}^{*}=\frac{\left(a\varphi +\theta \lambda \right)\Delta^{R}}{\varphi p\left(1-\lambda \right)}$*.*

Proposition 2, establishes that when the marginal profit of the manufacturer exceeds that of the online retailer, the manufacturer’s interest rate is zero. This is because the manufacturer enjoys a strong advantage of marginal profit in the supply chain, and this produces an interest-free credit service.

Proposition 2 helps to establish the following lemma and corollaries.

Lemma 2.

Under the TCF mode, there exists the same threshold $\lambda_{m}=\frac{a\varphi }{\theta }$ with the benchmark scenario when the e-commerce platform offers the live-stream selling service to consumers.

The explanation for Lemma 2 resembles Lemma 1, and we therefore omit it here.

Corollary 3.

$r_{m}^{*}$ is decreasing in λ*.*

When the e-commerce platform’s commission rate increases, this may increase the burden for the online retailer, with the consequence that the manufacturer has to stimulate the online retailer into financing and ordering by decreasing the interest rate.

Corollary 4.

(i) $\frac{\partial q_{m}^{*}}{\partial \theta }>0$*,* $\frac{\partial q_{m}^{*}}{\partial \varphi }<0$; (ii) when $\Delta^{M}<\Delta^{R}$, if $\lambda <1-\sqrt{\frac{\theta c+a\varphi c}{\theta p}}$, then $\frac{\partial q_{m}^{*}}{\partial \lambda }>0$, otherwise, we have $\frac{\partial q_{m}^{*}}{\partial \lambda }\leq 0$. (iii) when $\Delta^{M}\geq \Delta^{R}$, if $\lambda <1-\sqrt{\frac{\theta w+a\varphi w}{\theta p}}$, then $\frac{\partial q_{m}^{*}}{\partial \lambda }>0$, otherwise, we have $\frac{\partial q_{m}^{*}}{\partial \lambda }\leq 0$.

The explanation for Corollary 4 resembles Corollary 2 and we therefore omit it here.

### 4.3. E-commerce platform financing mode

In this mode, the e-commerce platform initially decides the financing interest rate $r_{e}$ for the online retailer at the beginning of the sales season, and then gives the live-stream investment $t_{e}$; after financing, the online retailer finally determines its ordering quantity $q_{e}$ before paying the manufacturer $wq_{e}$. At the end of the sales season the e-commerce platform can obtain the commission $\lambda p\min\left(q_{e},\;d_{e}\right)$ and the financing revenue $wr_{e}q_{e}$ from the online retailer, and meanwhile takes on an expected live-stream cost $\varphi t_{e}p\min\left(q_{e},\;d_{e}\right)$ with the online retailer yielding the remaining profit $\left(1-\lambda \right)p\min\left(q_{e},\;d_{e}\right)-wr_{e}q_{e}$. We therefore express the optimization problems of the e-commerce platform and well-funded retailer in Eqs 8, 9:


(8) $$\begin{array}{c} \underset{r_{e},\;t_{e}}{\max}E\left[\Pi_{e}^{E}\left(r_{e},\;t_{e}\right)\right]=\underset{r_{e},\;t_{e}}{\max}E\left[p\left(\lambda -\varphi t_{e}\right)\min\left(q_{e},\;d_{e}\right)\right]\\ +wr_{e}q_{e}, \end{array}$$

(9) $$\underset{q_{e}}{\max}E\left[\Pi_{e}^{R}\left(q_{e}\right)\right]=\underset{q_{e}}{\max}\left\{\begin{array}{l} E\left[p\left(1-\lambda \right)\min\left(q_{e},\;d_{e}\right)\right]\\ -w\left(1+r_{e}\right)q_{e} \end{array}\right\}\;.$$

In accordance with Eqs 8, 9, we assert the following proposition.

Proposition 3.

Under the EPF mode:

(i) When $\Delta^{R}<\frac{\left(1-\lambda \right)\sqrt{\Omega }-3w\Gamma }{\Gamma }$, the e-commerce platform’s optimal interest rate and live-stream investment are $r_{e}^{*}=\frac{\left(1-\lambda \right)\sqrt{\Omega }-3w\Gamma -\Delta^{R}\Gamma }{2w\Gamma }$ and $t_{e}^{*}=\frac{\theta \lambda -a\varphi }{2\theta \varphi }+\frac{\left(1-\lambda \right)\sqrt{\Omega }-3w\Gamma -\Delta^{R}\Gamma }{\varphi \left(\sqrt{\Omega }+p\Gamma \right)}$, the online retailer’s optimal ordering quantity is $q_{e}^{*}=\frac{\left(3p\Gamma -\sqrt{\Omega }\right)}{\varphi p\Gamma }\left\{\frac{\theta \lambda +a\varphi }{2}+\frac{\theta \Gamma \left[\left(1-\lambda \right)\sqrt{\Omega }-3w\Gamma -\Delta^{R}\Gamma \right]}{\sqrt{\Omega }+p\Gamma }\right\}$, where Γ=2θ−λθ+aφ and Ω=pΓ[pΓ+8θ(p−pλ+w)];

(ii) When $\Delta^{R}\geq \frac{\left(1-\lambda \right)\sqrt{\Omega }-3w\Gamma }{\Gamma }$, the e-commerce platform’s optimal interest rate and live-stream investment are $r_{e}^{*}=0$ and $t_{e}^{*}=\frac{\theta \lambda -a\varphi }{2\theta \varphi }$, the online retailer’s optimal ordering quantity is $q_{e}^{*}=\frac{\left(a\varphi +\theta \lambda \right)\Delta^{R}}{\varphi p\left(1-\lambda \right)}$.

Proposition 3 establishes that when the marginal profit of the online retailer is high, the e-commerce platform and retailer enjoy a strong advantage of profitability in the supply chain, and the e-commerce platform will therefore offer an interest-free credit service to the retailer.

Upon the basis of Proposition 3, we assert the following lemma.

Lemma 3.

Under the EPF mode, there is the same threshold $\lambda_{e}=\frac{a\varphi }{\theta }-\frac{2{\left[\left(1-\lambda \right)\sqrt{\Omega }-3w\Gamma -\Delta^{R}\Gamma \right]}^{+}}{\sqrt{\Omega }+p\Gamma }$ when the e-commerce platform offers the live-stream service to consumers, and $\lambda_{e}<\lambda_{o}=\lambda_{m}$.

The explanation for Lemma 3 resembles Lemma 1 and we therefore omit it here.

## 5. Comparative analysis of TCF and EPF

In Section 4, we obtain the optimal strategies of supply chain enterprises without capital constraint and under the TCF and EPF modes. Upon the basis of the first three propositions, we assert the following proposition.

Proposition 4.

$t_{o}^{*}=t_{m}^{*}\leq t_{e}^{*}$.

Proposition 4 helps us to conclude that the e-commerce platform will provide the highest live-stream investment for consumers under the EPF mode. This applies because, under this mode, the e-commerce platform can obtain financing profit from the online retailer, and this increased profitability will stimulate the e-commerce platform, enabling it to extend the live-stream investment it offers to consumers.

In order to clearly compare and analyze the value of TCF and EPF modes, we then employ several numerical examples with the intention of further illustrating the analysis of optimal strategies and enterprise profits under the TCF and EPF modes. Without loss of generality, suppose that the parameters are, respectively, a=100, θ=10, φ=0.01, w=10 and c=5.

**Figure 3:**
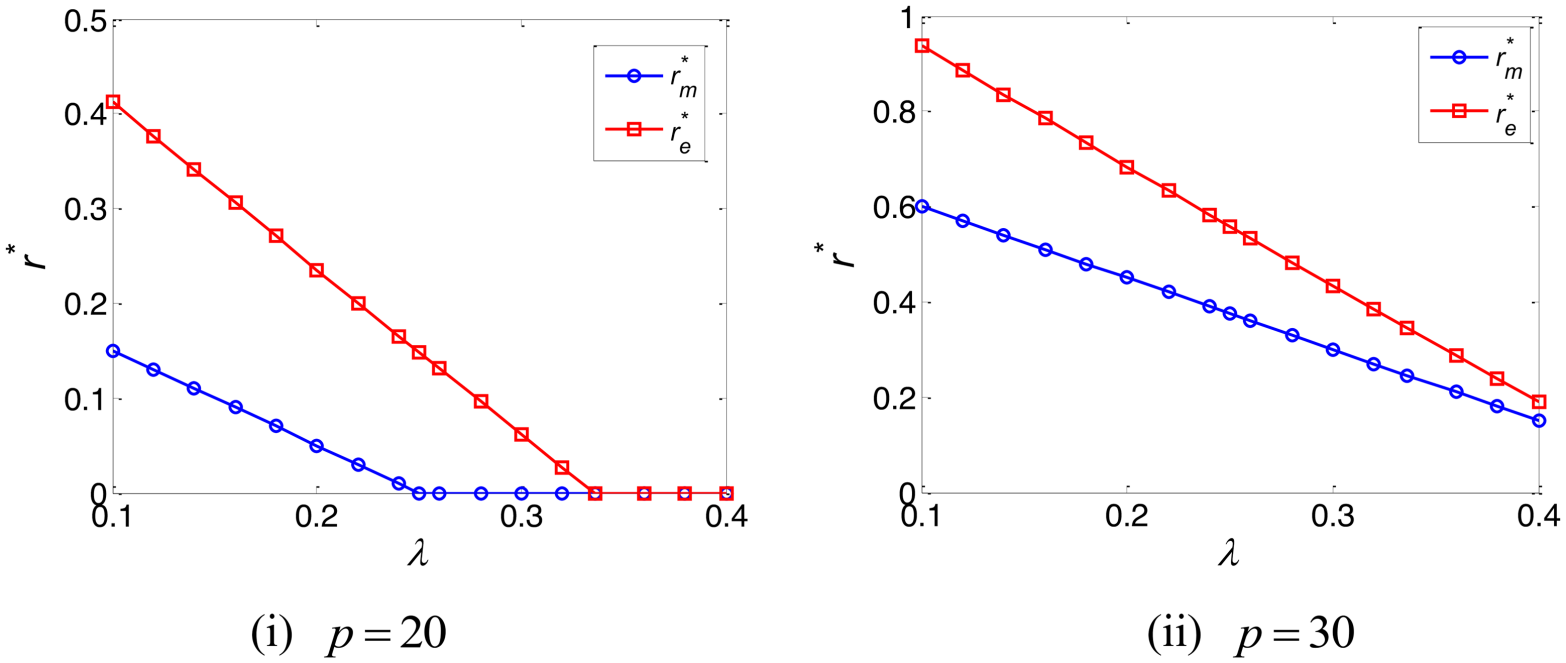
The optimal interest rate of the manufacturer and e-commerce platform.

The impact of the e-commerce platform’s commission rate on the optimal interest rate is depicted in Figure 3. This establishes that when the e-commerce platform’s commission rate is high and the online retailer’s marginal profit is low, optimal interest rates – under both TCF and EPF – are zero, a conclusion that accords with the results of Propositions 2 and 3.

**Figure 4:**
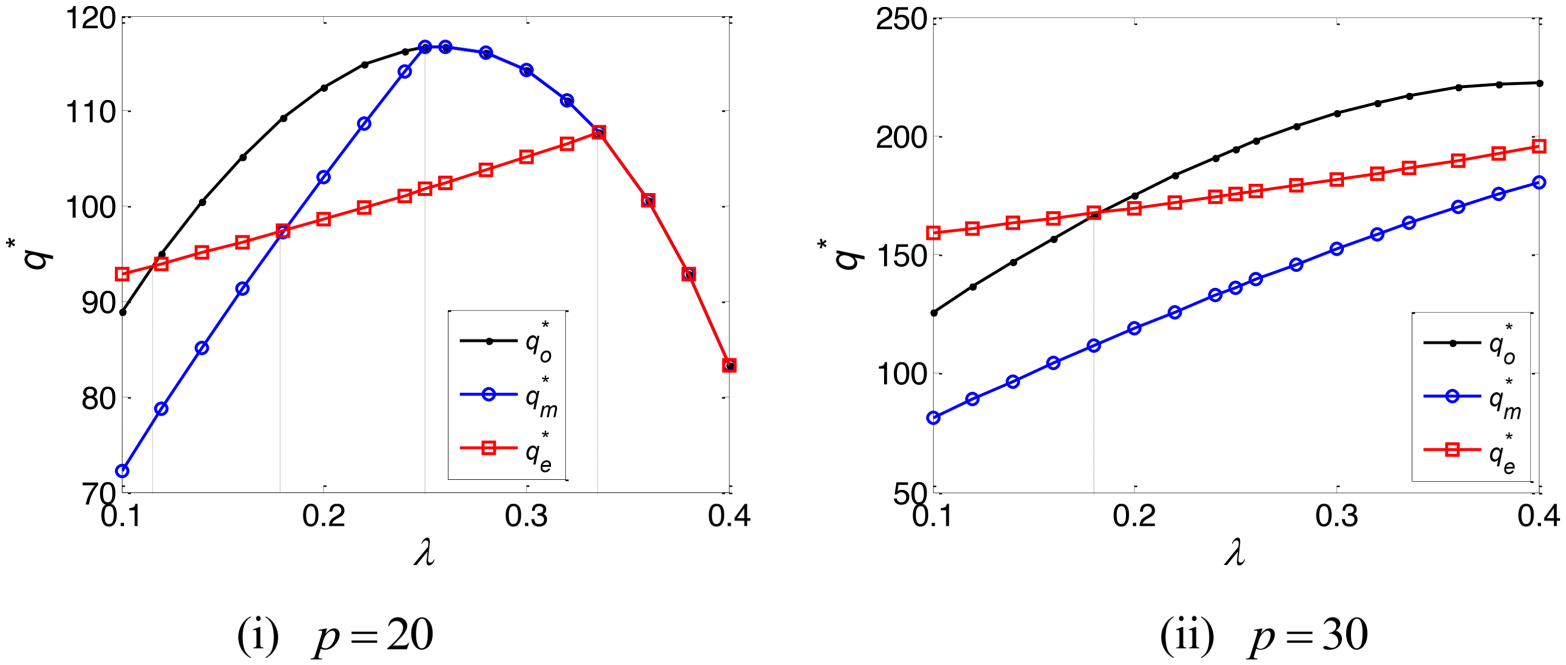
The optimal ordering quantity of the online retailer.

The impact of the e-commerce platform’s commission rate on the online retailer’s optimal ordering quantity is shown in Figure 4. This establishes two key conclusions: (i) When the e-commerce platform’s commission rate falls below a certain threshold, the online retailer’s optimal ordering quantity is, under EPF, in excess of that which would be evidenced without capital constraint; (ii) when the e-commerce platform’s commission rate is high and the online retailer’s marginal profit is low, the online retailer’s optimal ordering quantity is, under TCF, larger than that which would be evidenced under EPF. In all other respects, its optimal ordering quantity under EPF is higher. Figure 4 demonstrates how the EPF mode can, under certain conditions, stimulate the online retailer into ordering.

**Figure 5:**
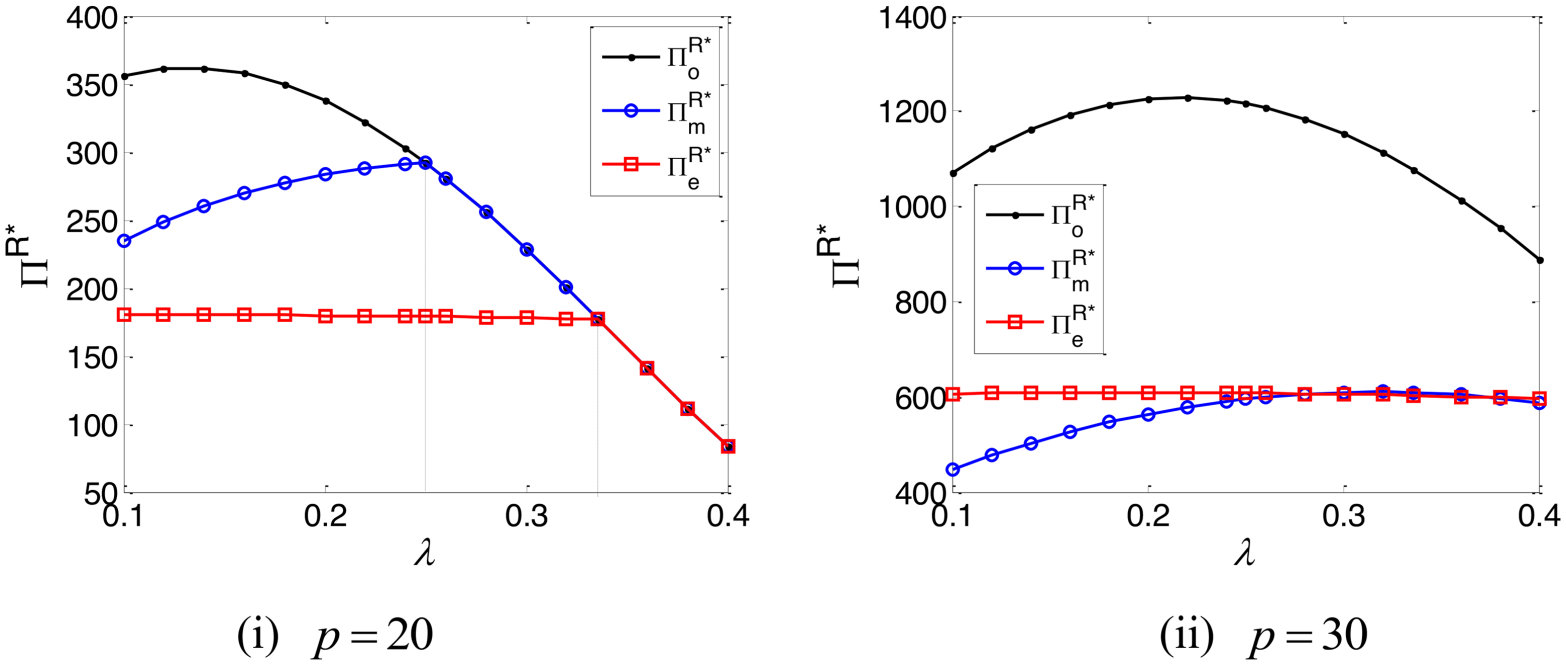
The profit of the online retailer.

The impact of the e-commerce platform’s commission rate on the online retailer’s profit is shown in Figure 5. This establishes that when the e-commerce platform’s commission rate is low and the online retailer’s marginal profit is high, the online retailer’s profit is, under EPF, larger than that which would apply under TCF; in all other respects, it can obtain the higher profit under TCF. It demonstrates that EPF is the optimal choice for the online retailer with a high marginal profit, while TCF is the optimal choice for the online retailer with a low marginal profit.

**Figure 6:**
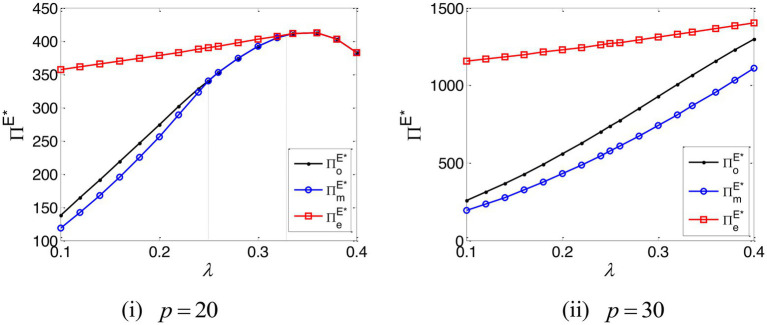
The profit of the e-commerce platform.

**Figure 7:**
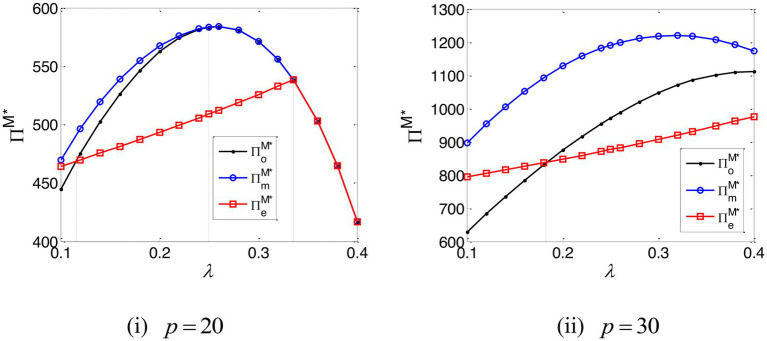
The profit of the manufacturer.

The impact of the e-commerce platform’s commission rate on the profit of the e-commerce platform and the manufacturer are, respectively, set out in Figures 6, 7. They establish that the e-commerce platform always prefers EPF, while the manufacturer always orientates toward TCF. In addition, when the e-commerce platform’s commission rate is low enough, EPF can benefit the manufacturer; TCF, however, cannot bring the extra yield for the e-commerce platform.

**Figure 8:**
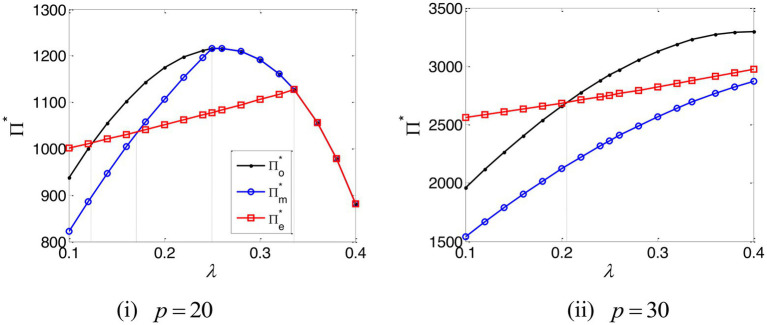
The total profit of the entire supply chain.

The impact of the e-commerce platform’s commission rate on the total profit of the supply chain is depicted in Figure 8. This demonstrates that when the e-commerce platform’s commission rate falls below a certain threshold, the total profit of the supply chain, under EPF, is in excess of that which would be evidenced without capital constraint. Meanwhile, when the e-commerce platform’s commission rate is high and the online retailer’s marginal profit is low, the total profit of supply chain, under TCF, exceeds that which would otherwise be demonstrated under EPF; in all other instances, the total profit of the supply chain under EPF is higher. Figure 8 demonstrates that while the EPF mode can, under certain conditions, create the added-value for the entire supply chain, TCF cannot do this.

## 6. Discussion

Live-stream selling is rapidly becoming a key marketing tool in e-commerce platforms, especially after the COVID-19 outbreak (Valaskova et al., 2021; Hao and Yang, 2022; Pan et al., 2022). According to a report on China’s live-stream industry, by the end of 2021, the number of live-stream users in China has reached 635 million, accounting for 61.5% of total Internet users, with 464 million e-commerce live-stream users (Hilvert-Bruce et al., 2018; Wongkitrungrueng and Assarut, 2018; Park and Lin, 2020; Dabija et al., 2022). As a result, live-stream selling became a multibillion-dollar business in China (Zhang et al., 2022). In 2020, Facebook started live-stream selling on its platforms. Walmart made live-stream selling on the TikTok. Other platforms, such as TalkShopLive, CommentSold, Brandlive, have vied to join live-stream commerce (Hao and Yang, 2022; Pan et al., 2022). In line with the existing literature (Hao and Yang, 2022; Pan et al., 2022; Zhang et al., 2022), our study also finds that there exists the threshold when the e-commerce platform offers the live-stream selling service to consumers. This paper helps us to conclude that the e-commerce platform will provide the highest live-stream investment for consumers under the EPF mode. This applies because, under this mode, the e-commerce platform can obtain financing profit from the online retailer, and this increased profitability will stimulate the e-commerce platform, enabling it to extend the live-stream investment it offers to consumers.

The majority of related research about supply chain financing shows that the internal financing mode (e.g., trade credit financing, logistics financing and platform financing) can encourage the capital-constrained retailer to increase the ordering quantity (Dada and Hu, 2008; Kouvelis and Zhao, 2016; Huang et al., 2019; Yan et al., 2022). Our study also obtain the similar conclusion. When the e-commerce platform’s commission rate falls below a certain threshold, the online retailer’s optimal ordering quantity is, under EPF, in excess of that which would be evidenced without capital constraint. When the e-commerce platform’s commission rate is high and the online retailer’s marginal profit is low, the online retailer’s optimal ordering quantity is, under TCF, larger than that which would be evidenced under EPF. In all other respects, its optimal ordering quantity under EPF is higher. These results demonstrate how the EPF mode can, under certain conditions, stimulate the online retailer into ordering. This establishes that when the e-commerce platform’s commission rate is low and the online retailer with high marginal profit can obtain larger profit under EPF than TCF.

From the perspective of fund providers, the e-commerce platform and the manufacturer both would like to offer financing service for the online retailer, which corresponds with the existing literature slightly (Cai et al., 2014; Cao and Yu, 2018; Gao et al., 2018; Liu et al., 2019; Wang et al., 2022; Yan et al., 2022). It shows as well the multiple service could actually bring the more profit for the e-commerce platform or the manufacturer, which will motivate the e-commerce platform or the manufacturer to offer financing service for the e-retailer. In addition, from the perspective of cross impact, when the e-commerce platform’s commission rate is low enough, EPF can benefit the manufacturer; TCF, however, cannot bring the extra yield for the e-commerce platform. This finding differs from the related literature (Wang et al., 2022; Yan et al., 2022).

Considering the total profit of the supply chain, our study find that when the e-commerce platform’s commission rate falls below a certain threshold, under EPF, is in excess of that which would be evidenced without capital constraint. Meanwhile, when the e-commerce platform’s commission rate is high and the online retailer’s marginal profit is low, the total profit of supply chain, under TCF, exceeds that which would otherwise be demonstrated under EPF; in all other instances, the total profit of the supply chain under EPF is higher. This demonstrates that while the EPF mode can, under certain conditions, create the added-value for the entire supply chain, TCF cannot do this. This is because that EPF mode can better weaken the double marginal effect among the supply chain members than TCF mode. Similarly, some classical research has demonstrated that TCF can overcome the external financing mode (e.g., bank financing mode) (Burkart and Ellingsen, 2004; Cai et al., 2014; Chen, 2015; Cao and Yu, 2018; Wang et al., 2018; Fu et al., 2021)., but in my study, we find EPF is better than TCF in most cases, which is a valuable new conclusion different from the existing literature. It also explains why more and more e-commerce platforms are carrying out financing services in many areas. So let us go to some actual real examples, Alibaba and Amazon have been carrying out financing services for capital-constrained e-retailers since 2010 and 2011, respectively. JingDong Mall offered over 1 billion RMB within the first month after starting “JingXiaoDai.” ZhaoGang Netcom launched “Pangmao Baitiao” for its e-retailers in 2014. The transaction volume using this financing service rose from 227 million RMB in 2015 to 2,790 million RMB in 2016, and to 5,913 million RMB in 2017 (Liu et al., 2019; Jena et al., 2022; Yan et al., 2022).

In taking into account the fact that the EPF mode can improve the profit of the entire supply chain, the manufacturer, e-commerce platform and online retailer should consider establishing the basis for enhanced cooperation among themselves by, for example, considering the drafting and application of contracts (e.g., a revenue-sharing contract).

## 7. Conclusion

This paper considers a supply chain that consists of a manufacturer, an e-commerce platform and an online retailer with capital constraints. In addition, we also take into account a situation where the e-commerce platform offers live-stream selling service to consumers. In our study, the manufacturer and e-commerce platform can both provide a financing service to the online retailer. On this basis, we study the optimal decisions of three enterprises undertaken under the TCF and EPF modes. Meanwhile, we take the decisions and profits of all enterprises without capital constraint as the benchmark, and compare how TCF and EPF impact on financing and operational decisions. Important conclusions will now be summarized.

First, the e-commerce platform’s commission rate and the marginal profits of the manufacturer and online retailer are the key factors that affect the enterprises’ financing and operational decisions. There exists the threshold when the e-commerce platform offers the live-stream selling service to consumers under three scenarios. However, the access threshold is lower under EPF mode, resulting to a larger live-stream investment.

Second, when the e-commerce platform’s commission rate is sufficiently low, the retailer’s ordering quantity is, under the EPF mode, greater than that which would apply without capital constraint - in other words, the EPF mode can stimulate the online retailer into ordering. In addition, when the retailer’s marginal profit is high and the e-commerce platform’s commission rate is low, the online retailer can obtain higher profit under the EPF mode; in all other respects, the TCF mode will produce a higher profit for the retailer.

Third, the e-commerce platform can obtain the highest profit under the EPF mode, while the TCF mode will result in the highest profit for the manufacturer.

Finally, when the platform’s commission rate is low and falls below a certain threshold, the total profit of the supply chain, when conceived under the EPF mode, exceeds that which can be obtained when the retailer has sufficient funds but the TCF mode cannot create value-added income that benefits the entire supply chain.

A number of useful managerial implications that can be applied to e-commerce supply chain partners during the development of financing and operational strategies can be derived from the Stackelberg game model and associated theoretical analyses.

First, the online retailer with capital constraint should seriously consider its marginal profit and the e-commerce platform’s commission rate when choosing the financing mode. The EPF mode should only be selected when the retailer’s marginal profit is high and the e-commerce platform’s commission rate is low; in all other instances, the TCF mode should be selected.

Second, the manufacturer and e-commerce platform should both actively provide a financing service for the online retailer with capital constraint, as this will create an additional profit that will benefit them both. In addition, in operating under the EPF mode, the e-commerce platform should extend the live-stream investment that it provides to consumers. In order to attract retailers who have selected the EPF mode, the e-commerce platform may adapt various commission rates to different scenarios.

Third, in taking into account the fact that the EPF mode can improve the profit of the entire supply chain, the manufacturer, e-commerce platform and online retailer should consider establishing the basis for enhanced cooperation among themselves by, for example, considering the drafting and application of contracts (e.g., a revenue-sharing contract).

Our work opens up several avenues for future research. First, we assume that information is shared symmetrically among enterprises. In reality, however, the e-commerce platform sometimes possesses more precise information and more extensive data than other partners, and this provides it with more far-reaching insight into*, inter alia,* market demand and conditions. Accordingly, it would be worthwhile to identify how optimal financing and operational strategies can be developed under the condition of asymmetrical information. Second, this study only considers single-channel financing, and dual-channel financing remains as an avenue of enquiry that still needs to be explored and developed.

## Data availability statement

The original contributions presented in the study are included in the article/Supplementary material, further inquiries can be directed to the corresponding authors.

## Author contributions

SH: methodology, formal analysis, writing-original draft, and funding acquisition. BD: writing-review and editing. Z-PF: funding acquisition, supervision, and validation. ZL: writing-review and editing. All authors contributed to the article and approved the submitted version.

## Conflict of interest

The authors declare that the research was conducted in the absence of any commercial or financial relationships that could be construed as a potential conflict of interest.

## Publisher’s note

All claims expressed in this article are solely those of the authors and do not necessarily represent those of their affiliated organizations, or those of the publisher, the editors and the reviewers. Any product that may be evaluated in this article, or claim that may be made by its manufacturer, is not guaranteed or endorsed by the publisher.
