# Erratum: E-commerce platform financing versus trade credit financing: financing mode selection for online retailer considering live-stream selling in China

**DOI:** 10.3389/fpsyg.2023.1207188

**Published:** 2023-05-04

**Authors:** Frontiers Production Office

**Affiliations:** Frontiers Media SA, Lausanne, Switzerland

**Keywords:** capital-constrained, e-commerce platform financing, online retailer, trade credit financing, live-stream selling

Due to a publishing error, the Editor's name was incorrectly written as “Marco Chjriatti” and the Editor's affiliation was incorrectly stated as “Chengdu University, China.” The name should instead be written as “Raffaele Filieri” and the affiliation should be stated as “Audencia Business School, France.”

The publisher apologizes for this mistake. The original version of this article has been updated.

