# Supplementary material for: E-commerce platform financing versus trade credit financing: Financing mode selection for online retailer considering live-stream selling in China
Source: Front Psychol. 2023 Jan 10;13:1078369. doi: 10.3389/fpsyg.2022.1078369 (PMC9871903; doi:10.3389/fpsyg.2022.1078369)
Supplement: Supplementary file 1 [file Data_Sheet_1.doc]

**Appendix**

**Proof of Proposition 1**

Equation (4) establishes that we can obtain $q_{o}^{*}=2\left(a+\theta t_{o}\right)\left[1-\frac{w}{p\left(1-\lambda\right)}\right]$ from the first-order condition of $\frac{\partial E\left[\Pi_{o}^{R}(q_{o})\right]}{\partial q_{o}}=0$. Since $\frac{\partial^{2}E\left[\Pi_{o}^{R}(q_{o})\right]}{\partial q_{o}^{2}}=-\frac{p\left(1-\lambda\right)}{2\left(a+\theta t_{o}\right)} <0$, $q_{o}^{*}$ is the unique optimal solution.

Substituting $q_{o}^{*}$ into Equation (3) we reflect, in accordance with Equation (3), that we can obtain from the first-order condition of $\frac{\partial E\left[\Pi_{o}^{E}(t_{o})\right]}{\partial t_{o}}=0$. This applies because , $t_{o}^{*}$ is the unique optimal solution. Accordingly, Proposition 1 is proven.

**Proof of Lemma 1**

By Proposition 1, we can obtain the lower limit threshold from . Hence, Lemma 1 holds.

**Proof of Corollary 1**

According to Proposition 1, we have $\frac{\partial t_{o}^{*}}{\partial\theta}=\frac{a}{2\theta^{2}}>0$, , . Hence, Corollary 1 holds.

**Proof of Corollary 2**

According to Proposition 1, (i) we have and . (ii) Since , when , then $\frac{\partial q_{o}^{*}}{\partial\lambda}>0$, otherwise, we have $\frac{\partial q_{o}^{*}}{\partial\lambda}\leq0$. Hence, Corollary 2 holds.

**Proof of Proposition 2**

According to Equation (7), we can obtain $q_{m}^{*}=2\left(a+\theta t_{m}\right)\left[1-\frac{w\left(1+r_{m}\right)}{p\left(1-\lambda\right)}\right]$ from the first-order condition of $\frac{\partial E\left[\Pi_{m}^{R}(q_{m})\right]}{\partial q_{m}}=0$. This applies because $\frac{\partial^{2}E\left[\Pi_{m}^{R}(q_{m})\right]}{\partial q_{m}^{2}}=-\frac{p\left(1-\lambda\right)}{2\left(a+\theta t_{m}\right)} <0$, $q_{m}^{*}$ is the unique optimal solution.

Substituting $q_{m}^{*}$ into Equation (6) we reflect, in accordance with Equation (6), that we can obtain from the first-order condition of $\frac{\partial E\left[\Pi_{m}^{E}(t_{m})\right]}{\partial t_{m}}=0$. This applies because , $t_{m}^{*}$ is the unique optimal solution.

Substituting $q_{m}^{*}$ and $t_{m}^{*}$ into Equation (5) we reflect, in accordance with Equation (5), that we can obtain $r_{m}^{*}=\frac{\Delta^{R}-\Delta^{M}}{2w}$ from the first-order condition of $\frac{\partial E\left[\Pi_{m}^{M}(r_{m})\right]}{\partial r_{m}}=0$. Because , $r_{m}^{*}$ is the unique optimal solution. However, because $r_{m}^{*}\geq0$, we observe that when $\Delta^{M}<\Delta^{R}$, then $r_{m}^{*}=\frac{\Delta^{R}-\Delta^{M}}{2w}$, otherwise, $r_{m}^{*}=0$. Hence, Proposition 2 is proven.

**Proof of Lemma 2**

The proof is similar to Lemma 1, and is accordingly not repeated here.

**Proof of Corollary 3**

According to Proposition 2, we have $\frac{\partial r_{m}^{*}}{\partial\lambda}=-\frac{p}{2w}<0$. Hence, Corollary 3 holds.

**Proof of Corollary 4**

According to Proposition 1, (i) we have and . (ii) When $\Delta^{M}<\Delta^{R}$, we have , so we can know that if , then $\frac{\partial q_{m}^{*}}{\partial\lambda}>0$, otherwise, we have $\frac{\partial q_{m}^{*}}{\partial\lambda}\leq0$. (iii) When $\Delta^{M}\geq\Delta^{R}$, we have , so we can know that if , then $\frac{\partial q_{o}^{*}}{\partial\lambda}>0$, otherwise, we have $\frac{\partial q_{o}^{*}}{\partial\lambda}\leq0$. Hence, Corollary 4 holds.

**Proof of Proposition 3**

According to Equation (9), we can obtain $q_{e}^{*}=2\left(a+\theta t_{e}\right)\left[1-\frac{w\left(1+r_{e}\right)}{p\left(1-\lambda\right)}\right]$ from the first-order condition of $\frac{\partial E\left[\Pi_{e}^{R}(q_{e})\right]}{\partial q_{e}}=0$. Since $\frac{\partial^{2}E\left[\Pi_{e}^{R}(q_{e})\right]}{\partial q_{e}^{2}}=-\frac{p\left(1-\lambda\right)}{2\left(a+\theta t_{e}\right)} <0$, $q_{e}^{*}$ is the unique optimal solution.

Substituting $q_{e}^{*}$ into Equation (8), according to Equation (8), we can obtain from the first-order condition of $\frac{\partial E\left[\Pi_{e}^{E}(t_{e})\right]}{\partial t_{e}}=0$. Since , $t_{e}^{*}$ is the unique optimal solution.

Substituting $t_{e}^{*}$ into Equation (8), in accordance with Equation (8), establishes that we can obtain $r_{e}^{*}=\frac{\left(1-\lambda\right)\sqrt{\Omega}-3w\Gamma-\Delta^{R}\Gamma}{2w\Gamma}$ from the first-order condition of . It is easy to know that $\frac{\partial t_{e}^{*}}{\partial r_{e}}>0$, $\frac{\partial^{2}t_{e}^{*}}{\partial r_{e}^{2}}<0$, $\frac{\partial q_{e}^{*}}{\partial r_{e}}<0$ and $\frac{\partial^{2}q_{e}^{*}}{\partial r_{e}^{2}}<0$. Therefore we have established , $r_{e}^{*}$ is the unique optimal solution. However, because $r_{e}^{*}\geq0$, we have when $\Delta^{R}<\frac{\left(1-\lambda\right)\sqrt{\Omega}-3w\Gamma}{\Gamma}$, then $r_{e}^{*}=\frac{\left(1-\lambda\right)\sqrt{\Omega}-3w\Gamma-\Delta^{R}\Gamma}{2w\Gamma}$, where and $\Omega=p\Gamma\left[p\Gamma+8\theta\left(p-p\lambda+w\right)\right]$, otherwise, $r_{e}^{*}=0$. Hence, Proposition 3 is proven.

**Proof of Lemma 3**

According to Proposition 3, when $\Delta^{R}\geq\frac{\left(1-\lambda\right)\sqrt{\Omega}-3w\Gamma}{\Gamma}$, we have , so we can obtain the lower limit threshold . When $\Delta^{R}<\frac{\left(1-\lambda\right)\sqrt{\Omega}-3w\Gamma}{\Gamma}$, we have $\left(1-\lambda\right)\sqrt{\Omega}-3w\Gamma-\Delta^{R}\Gamma>0$, so we can obtain the lower limit threshold from $t_{e}^{*}>0$. Hence, Lemma 3 is proven.

**Proof of Proposition 4**

According to Propositions 1-3 and Lemmas 1-3, it is easy to know $t_{o}^{*}=t_{m}^{*}\leq t_{e}^{*}$. Hence, Proposition 4 is proven.
